# User requirements for the design of smart homes: dimensions and goals

**DOI:** 10.1007/s12652-021-03651-6

**Published:** 2022-05-30

**Authors:** Michaela R. Reisinger, Sebastian Prost, Johann Schrammel, Peter Fröhlich

**Affiliations:** 1 Austrian Institute of Technology, Giefinggasse 4, 1210 Vienna, Austria; 2 Newcastle University, Newcastle Upon Tyne, NE1 7RU UK

**Keywords:** Smart home, Home control, User requirements, Design implications

## Abstract

The ‘Smart Home’ is a strongly technology-driven field. While user-centered requirements have been reported for specific features, a considerable gap persists for design based on an everyday home context and the social and emotional nature of the home. To address this, we present a user-centered design process to question and expand narrow framings of energy-efficiency and smart control and consider the richness and variety of the domestic context as design space for smart homes. Our three-step investigation employs cultural probing, participatory design fiction, and focus groups to progress from the home context “as-is” towards a blending of values with technological responses. Our findings highlight the home as a complex construct imbued with organically grown practices and individual and collective needs, values, and emotions. Based on empirical, real-user data we present features and system expectations that address this multifaceted overall picture. This paper advises the design process of future smart home solutions in three facets: first, we discuss the value of the design process applied in this study and future possibilities to expand. Second, we show *design dimensions*, namely time, space, relations, individual factors, and values that allow design for a heterogeneity of users and situations. Third, we derive specific *design goals* to highlight directions of smart home system design: design for control, low effort, integration, evolvability, identity, sociability, and benefits.

## Introduction

“Smartness” is infectious: it is extending above and beyond specific devices, as well as contexts—including the home (Taylor et al. 2007; Wilson et al. 2015). Smart homes have become technologically available and feasible (De Silva et al. 2012; Sciuto and Nacci 2014; Sadikoglu-Asan 2020), and the interest in smart homes in research and industry is growing exponentially (Solaimani et al. 2013b; Wilson et al. 2015), yet, their appeal to the general population is limited (Taylor et al. 2007; Marikyan et al. 2019): while the COVID-19 pandemic might accelerate technology adaption in the home (Maalsen and Dowling 2020), perceived and potential benefits continue to be incongruent and adoption and diffusion rates remain low (Marikyan et al. 2019). The “socio-economic and technological constellation” (Friedewald et al. 2005, p. 236) necessary for their success has—still—not been met. A key issue is that most visions of smart homes are not based on the lived realities in actual homes. The term “smart home” very rarely refers to what *home* means as an inhabited space but rather to its technology (Innocenti 2017). Likewise, smart home design does frequently not address the perspective of actual users and everyday life (Strengers 2016): it is questionable whether depicted sterile, empty spaces at odds with home realities (Wilson et al. 2015) are able to comprehend the emotionally laden “invisible boundaries” that form the home context (Davidoff et al. 2006). Highly individual, diverging combinations of items pose another home-specific challenge to the design of smart home systems (Davidoff et al. 2006), as does the investment and scope they represent—“instrumenting” a person’s whole living environment (Mennicken and Huang 2012a). Most smart home designs also implicitly envision “resource man” (white, male, middle-class) with a keen interest in monitoring and optimizing energy consumption—a very specific subgroup of individuals found in homes (Strengers 2014). Investigating the relationship between user and home has only just begun (p. ex. Sadikoglu-Asan 2020).

Yet, the home context could specifically benefit from smart home solutions: inhabitants spend a large amount of time within their homes, and home activities have great potential to evolve and enhance themselves (Friedewald et al. 2005)—which is particularly evident in the demands a pandemic such as COVID-19 places on the home and the changes homes have undergone in the last year (Maalsen and Dowling 2020). Smart home design could facilitate daily activities, support managing specific needs and domestic energy demand, meeting the challenges of digitalization, and enabling inhabitants’ socialization (Friedewald et al. 2005; Wilson et al. 2015). Smart home technology might even add new aspects with which a home can be imbued with identity and personality, the act which makes a house a home (Innocenti 2017).

In this paper, we present a study that explores the home context through three user-centered steps. Viewing the home as an individual everyday environment we identify design directions to achieve a closer fit between design and context.

## Related work

To explore users’ perspectives of smart homes, we will first look at the home context, what constitutes a ‘smart home’, and previous research engaging potential inhabitants.

### The home context

The home is a complex place: it is inhabited by one or multiple users, who are subject to change. The technology they use, their relationships and responsibilities evolve over time—especially in multiple user homes, which are characterized by a shared ownership of tasks, individual levels of comfort, task completion, and competing needs.

Necessary negotiation over time employs available surfaces for information sharing, such as fridge doors and wall displays (Taylor et al. 2007). Homes are built on specific values like emotional connection, self-expression, and identity and therefore feature spaces of importance (e.g. the kitchen as the hub of home) (Davidoff et al. 2006). Users’ relationships with domestic technologies have been shown to focus on emotional attribution and attachment, which creates the feeling, or quality of home (Mäyrä et al. 2006). Home technologies have to recognize such emotional significances to avoid crossing “invisible value lines” and provide assistive tasks without challenging the identity of the user or their feeling of control (Davidoff et al. 2006).

Home behavior has been noted to be unstructured (De Silva et al. 2012). While that is largely true, domestic activities do feature routine practices revolving around timeframes, like morning routines or arriving-home practices. Once established, these routines are highly specific yet evolving procedures (Coutaz et al. 2010). Events which cannot be routinized (e.g. activities with varying details), unexpected exceptions (e.g. illnesses) and other deviations can be experienced as stressful as well as positive: While a sudden sickness calls for improvisation and flexibility to accomplish necessary tasks (Davidoff et al. 2006), other actions only retain their meaning while not firmly embedded in a routine (e.g. spontaneous messages, Taylor et al. 2007). Accounting for routines as well as enabling deviations is most important to smart home systems (Davidoff et al. 2006; Coutaz et al. 2010). Routines can be difficult to automate (Davidoff et al. 2010) and rarely translate into predefined scenarios (Davidoff et al. 2006), but can be realized through customizable rules/scenarios (Woo and Lim 2015).

### Smart homes in theory: features, expectations and motivations

According to Balta-Ozkan et al. (2013a), a smart home is defined by four key aspects: a *communication network*, which connects *sensors* (and devices); *intelligent controls* for system management, and *smart features* that respond to user-, sensor- or system (data) input. Devices in such a smart home can be controlled, accessed and monitored remotely. Consistent with this definition, many studies focus on features that make a home ‘smart’ (Koskela and Väänänen-Vainio-Mattila 2004; De Silva et al. 2012; Balta-Ozkan et al. 2013a)—for example by elucidating control preferences (Kühnel et al. 2011; Deloitte Consulting and TU München 2015), energy feedback preferences (Karjalainen 2011), energy monitoring (Murtagh et al. 2014), and acceptance/perceptions of smart homes (Balta-Ozkan et al. 2013b, 2014; Zhai et al. 2014). User-centered definitions of “smartness” in the home context often take a different, less feature-based approach, for example highlighting a good fit with everyday-life and a substantial improvement of the status-quo (i.e. being faster or better, not merely convenient; Mennicken and Huang 2012a). In the following, we present relevant studies investigating user expectations and anticipations regarding smart homes and related technology.

A popular aspect is *home control*, i.e. the central control of appliances and devices. Previous studies identified different objects most important for users in different countries (Kühnel et al. 2011; Zhai et al. 2014; Deloitte Consulting and TU München 2015)—alarm and childcare systems, temperature and humidity control, smart cleaning, energy management, lights and TV being mentioned most, followed by blinds, plugs, switches as well as remote (smartphone) access. This aspect of control seeks to improve convenience through automation—a positive effect that has been noted to be counteracted by perceived or actual loss of control (Mennicken and Huang 2012b).

Frequently, smart home visions focus on *energy feedback and energy management* (Balta-Ozkan et al. 2013b; Marikyan et al. 2019)—placing the purpose and potential of the smart home within the realm of energy efficiency and employing a deeply instrumental view of smart homes (Wilson et al. 2015). A focus on “the *sustainable* smart home” has been discussed to contradict the need of “the *desirable* smart home”, which enhances comfort, convenience, and security through new household practices, which consume additional electricity (Jensen et al. 2018b, p. 355; Strengers et al. 2020). Consumption (feedback) is a highly value-laden subject, covering issues of trust, privacy, environmental concern, and financial savings: In a study conducted in the UK, Germany, and Italy, participants expressed a lack of trust in power companies as well as concern about privacy and security when using a smart home system (Balta-Ozkan et al. 2013b, 2014). Only 10% of German respondents would trust their energy provider with their data (Deloitte Consulting and TU München 2015). Interest in energy management has been shown to be based on environmental concern and/or on the wish to reduce costs (Karjalainen 2011; Balta-Ozkan et al. 2014). UK participants noted an increased agency through knowledge, while German and Italian participants valued transparent information about energy use most (Balta-Ozkan et al. 2014). Feedback preferences from Finland show interest in consumption breakdown to individual appliances and comparisons with one’s own prior consumption (Karjalainen 2011). UK households equipped with energy monitoring systems show a wide variation in their attempts to save energy, depending on whether and how the behaviors themselves were situated within their physical and social context, and whether saving behavior was ingrained before and independent of any use of energy monitoring systems (Murtagh et al. 2014). Energy consumption feedback can also be coupled with time-variable tariffs, which are generally promoted as aiding energy conservation and reducing costs but are often met with reservations, mainly focused on low potential savings, practicality, and the difficulty of behavior change within appointed slots (Karjalainen 2011; Balta-Ozkan et al. 2014; Prost et al. 2015).

*Communication features* in smart homes have largely been limited to machine-human communication (e.g. notifications about [system] events), even in cases where users were involved in developing new services (Coutaz et al. 2010). As argued earlier, the home is a place of social interaction and negotiation among humans. Features supporting such activities have, however, been rarely discussed in previous studies.

Regarding user’s *expectations* of smart homes, German participants expected increased comfort, sustainability and security, lower heating and energy costs, and fun, though 29% of respondents could not name any reasons for using a smart home (Deloitte Consulting and TU München 2015). UK participants saw potential benefits in increased quality of life and leisure time, support of energy conservation, energy savings (reduced costs, environmental benefit), and assisted living (Balta-Ozkan et al. 2013b). They were skeptical whether substantial savings could presently be achieved. UK and Italian participants also noted support for assisted living (Balta-Ozkan et al. 2014). In another UK study, smart home technology use was mostly motivated by the possibility of energy-saving and cost reduction (often coupled with a prior interest), by an interest in technology and home automation which would lead to an improved home control in the home (including convenience and comfort) and environmental protection (Hargreaves et al. 2018). In addition to the motivation of saving energy or money, Mencken and Huang (2012a) found participants invest in smart home technologies out of general interest (as a hobby) or to pursue the idea of “a modern home”, investigating smart home options in order to consider “the latest technology” when building their new home.

Users noted several *challenges* that currently prevent the mainstream adoption of smart homes. Chinese and European participants noted privacy, data security, cost, reliability, and reliance as major barriers (Balta-Ozkan et al. 2013b; Zhai et al. 2014; Deloitte Consulting and TU München 2015), as well as concerns about smart home technology being divisive and exclusive (Balta-Ozkan et al. 2013b). Further challenges included a lack of perceived benefits and fear of change (Deloitte Consulting and TU München 2015) as well as high perceived complexity and unfamiliarity with smart home technology (Taylor et al. 2007). Some of these issues, such as privacy and reliability, have been perceived as primarily technical issues and not been addressed from a user’s perspective. Thus, the key challenges for the design of user-home interactions lies in the realms of security, privacy and trust, usability and user-friendly design (Wilson et al. 2015).

### Smart homes in practice: approaches to domestication

Smart homes are very specific utopias that might or might not come true once people start living in them (Strengers 2016). The following studies, therefore, shift the focus from features, expectations, and motivations towards experiences with smart homes. Mencken and Huang (2012a) investigated this “real-world” process of domesticating smart technologies by involving individuals who were planning or building a smart home and those already living in smart homes, as well as smart home providers. They show that of four key phases—initial planning, preparing the technical infrastructure, iteration and configuration, and reaching (temporary) stability—planning, integrating, and iterating had a greater impact on participants than technology use when stability was reached. They also identified three distinctive roles within households: *Home technology drivers* who have a technical background and interact with the system as a hobby; *home technology responsibles*, who are invested but outsource technological functioning to professionals; and *passive users*, who are not actively engaged, but shape a smart home indirectly through evaluation. They note that tensions can arise between individuals of different roles. Similarly, a nine-month field trial of ten UK households showed smart homes to be “technically and socially disruptive” (Hargreaves et al. 2018, p. 127). Their study highlights the cognitive, practical and symbolic work necessary for integrating smart home technologies within a home, which is undertaken by different individuals. This rather high cost poses a challenge to smart home adaptation which is so far not facilitated by design.

Both studies note a need for support beyond making end-user configuration of interfaces palatable, including direct instruction and integration (e.g. tradespeople being familiar with systems and supporting their integration) but also demonstrating potential functionalities (Hargreaves et al. 2018) and supporting individuals in choosing technologies and iterating their use (Mennicken and Huang 2012a). Information like websites, manuals, and brochures, should, therefore, focus less on technical details and more on potential use in everyday life. Mennicken and Huang (2012a) also elaborate on different kinds of support for their user types: Technology drivers should have the possibility to access the complex side of smart homes, because supporting development as a hobby, for example by unifying languages and enabling sharing of code, would not only satisfy the needs of this group but could also minimize effects on other groups who would experience fewer malfunctions and less inconvenience. Support for the collaborative evolution of smart home technologies could on the other hand allow passive users to be included and heard without having to invest effort in managing the system itself. Including all users in home domestication is vital for enabling the shared creation of meaning within a space, which is denoted by the term “home” (Innocenti 2017).

Investigating initial and prolonged use, Hargreaves et al. (2018) found three domestication pathways for smart homes: In their study, *successful domestication* was achieved by abandoning more advanced features to effectively use smart technologies. *Precarious domestication* on the other hand was characterized by irregular use—too interesting to be abandoned but too high effort to consistently use. *Non-domestication*, i.e. the rejection of smart home technologies, occurred with individuals with little interest in technology, who saw smart home technologies as offering little to no benefit with an actual or potential negative impact on individuals (losing control), environment (greater energy use), or society (becoming lazy). These results are very much in line with Mennicken and Huang (2012a), who found that the lack of a clear vision of potential benefits and knowledge on what functionality to expect, hampers smart home domestication, especially in the planning phase. Lacking a sound basis for decision-making such as understanding the different options available made people without technological background feel powerless.

Further research shows that the perspective of smart home use and functionality could be enriched by not only demonstrating such functionalities—thereby limiting them to perceivable use-cases—but also adding perspectives on outcome and experience which allow participants to create and implement their own use-cases:

A 3-year qualitative study of 23 Australian households using smart home technologies explored the potential impact of desires on energy consumption and compared three distinct approaches (reason, ethics and aesthetics; Nelson and Stolterman 2000, 2003) to create three ‘smart home personas’: the *helper*, the *optimizer* and the *hedonist* (Jensen et al. 2018a). Their desires, the functional capability, the outcome of the smart home, and the aesthetic experiences derived from it, shape the expectations and experiences of living with smart home technology, both complementing and contrasting with each other. Their approach elucidates an angle to smart home design that does not try to problem-solve user-home interaction but exploring how user motivation can shape smart homes towards sustainability. In a second study, Jensen et al. (2018b) probed two Australian households living with smart home lighting technology over four weeks to explore a smart home vision that combined what is *desirable* (aesthetic experience associated with comfort) with what is *sustainable* (energy use). Their probes addressed energy efficiency not as a value (“to do the right thing”) but as a side-effect of a specific atmosphere of comfort (through the concept of *hygge*^1^). This focus on atmosphere and aesthetic appreciation did not only shape the use of technology in a sustainable manner but allowed participants to include all aspects of their home in these practices, including non-electrical material and existing activities.

Smart homes in theory show a well-developed focus on investigating specific functionalities yet lacking others as well as an overall view. While it elucidates features and topics to base our research on, it also shows that *“[…] a clear user-centric vision of smart homes is currently missing from a field being overwhelmingly ‘pushed’ by technology developers”* (Wilson et al. 2015, p. 464). Experiences from smart home domestication show that it is necessary and worthwhile to investigate how individuals are living their daily lives, how these lives are shaped or disrupted by smart home technologies, to include social practices, concepts of agency and materiality to understand the interaction of digital, material and human in everyday practice as well as the role of a smart home as an (inter)acting agent, which in turn also prompts reactions (and not only actions) from its inhabitants (Strengers 2016).

This paper, therefore, aims at providing design directions that include the everyday character and *context* of homes (Davidoff et al. 2006; Taylor et al. 2007) by combining expert and participant input in three phases. By that, we respond to user needs within the smart home (Coutaz et al. 2010) as well as address the challenges for smart home design (Solaimani et al. 2013a; Wilson et al. 2015) outlined above, enriching but also moving beyond the research of relevant features.

## Methods, procedure and materials

**Fig. 1:**
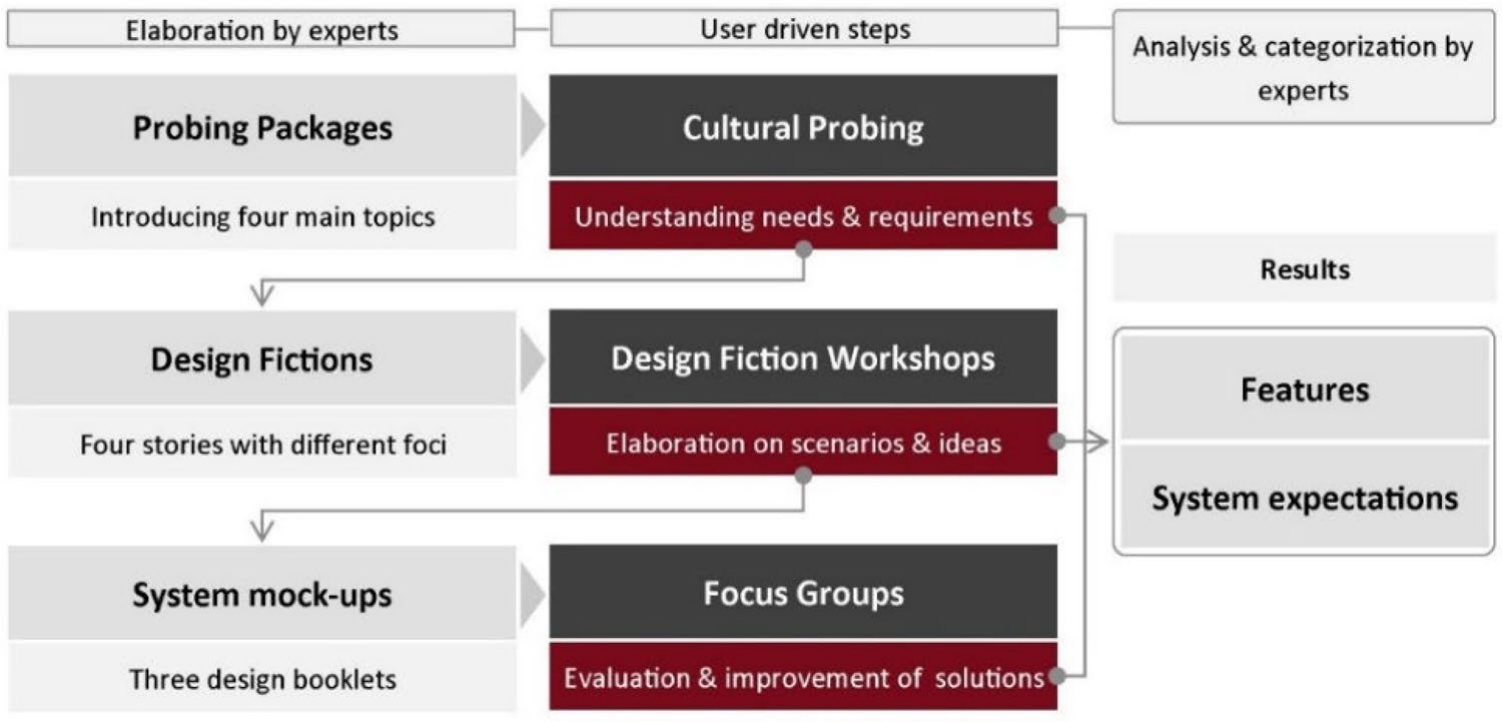
The overall process: light colored boxes (left, right) indicate input and elaboration by experts (researchers, designers), while dark colored boxes (middle) indicate user involvement

Our approach was divided into three main phases, each of which combined field-specific, scientific expertise, and participants’ input (Fig. 1). It started with open self-observations of everyday interactions and the home context and then moved via design scenarios to detailed design concepts. Each phase yielded results in the shape of features and system expectations, which were then analyzed as to their design implications.

The procedure for each phase is described in the sections below, addressing the following aspects:

1. Deepen the understanding of everyday life interactions through an initial screening questionnaire, cultural probing and follow-up interviews. The aim was to elucidate user values, basic home principles and specific context factors.
2. Develop smart home “design fictions” (fictional smart home narratives, see below) using the findings of the cultural probing phase and expounding on (alternative) features with users in design fiction workshops.
3. Design and evaluate smart home components through focus groups that discuss and evaluate design prototypes based on the workshop results.

This study was part of a larger research project, which accompanied individuals moving into a newly developing urban neighborhood. It was originally planned to capture existing routines through cultural probing well before participants move into their new homes, and to include them in design fiction workshops and focus groups through the first phase in their new living environment which would introduce several smart features. However, due to construction delays and legal concerns, we could only draw on a limited number of participants for the three phases described in this paper and consequently included individuals from an external user panel. There was no overlap between participants of different phases.

### Deepening the understanding of everyday life interactions

The first phase focused on understanding the practices of people in their everyday environment including routines in which they (potentially) interact with home technology. The goal was to identify design possibilities and opportunities that could support and enrich these interactions. The main method applied was cultural probing (Gaver et al. 1999), an in-depth measure typically using a small, carefully chosen sample. We employed screening and theoretical sampling to achieve a well-balanced user group:

The *online screening questionnaire* elucidated different aspects of participants’ lives, including living situations, experience with home technologies, as well as general attitudes and interests. We employed an external user panel and received 251 completed questionnaires, of which 213 expressed a willingness to participate in follow-up studies. The following set of criteria was used for recruiting participants for each study phase:

- Achieve gender balance as well as diversity in age, educational background and technology affinity.
- Include different types of households (e.g. individuals with and without children).
- Include participants with different experience levels with smart home technologies, including both positive and negative experiences with existing appliances.

While most participant characteristics could be reasonably controlled (Table 1), most screening respondents replied to be technologically and environmentally interested, which might not be representative of the broader population. Since participants were largely recruited from the screening questionnaire, these biases are also present within the study samples.

**Table 1:** Cultural probing participants

Gender	
Male	5
Female	5

Age group	
19–35	3
36–50	4
51+	3

Interest and commitment (very – more – less – not interested/committed)	
Technology	6 – 3 – 1 – 0
Environment	4 – 3 – 2 – 1
Community	4 – 1 – 2 – 3
Politics	3 – 3 – 1 – 3

Education	
Compulsory education	1
Apprenticeship	3
Vocational school	1
Secondary school	3
College/University	2

Living situation	
Single	2
Domestic partnership, children	3
Domestic partnership, no children	3
Single parent	2

*Cultural probing* is a method to gather data about people’s lives, values and thoughts to inspire and guide a subsequent design process (Gaver et al. 1999). *Probes* typically consist of artifacts (e.g. maps, postcards, camera) that are used by participants to record feelings and interactions related to a design problem. The main goal is to gather unsupervised responses over time. Our probe included a notebook, 20 action cards, materials to draw a map of their home and a single-use camera or photo-upload link. The diary-styled notebook contained prompts to document appliance use daily as well as eight photo tasks (e.g. “Please photograph technology that saves you time.”, adapted from Haines et al. 2007). The action cards focused on four home-related aspects, based on previous research (Sect. 2): energy use, home control, information, and communication and participation. Participants were asked to use two cards per day. Each included an action (e.g. “Please find appliances on stand-by and mark them on the map with the stickers provided.”) combined with an open-ended statement (“This appliance is usually on standby, because…”). Ten participants recorded their experiences for ten days, paying attention to different elements of their home-life. After a first analysis of their probe, each participant was invited for a follow-up interview, which first discussed their daily routines. Participants were asked why and when they use certain devices and how they create comfort and wellbeing. We then discussed each probing item (e.g. photo, card), with in-depth questions for items we found particularly interesting or unclear in the analysis of the probe.

### Development and elaboration of design fictions

Investigating scenarios of smart home interactions helps to understand the consequences and chances of such approaches. This phase of our research process, therefore, developed several fictional scenarios based on phase one, including for example real-life household routines, contexts for using spaces and objects within the home and emotions participants shared through probing material. We subsequently elaborated these fictional stories with potential users using the method of participatory design fiction (Grand and Wiedmer 2006).

*Design fictions* are a creative way to elaborate on speculative, but realistic visions of the future (Sterling 2005; Grand and Wiedmer 2006). They easily translate into design—originally conceived as a tool for designers, they have been used in a participatory manner with users (Prost et al. 2015). Design fictions are less task-oriented and wider in scope than more commonly used user scenarios (Blythe and Wright 2006). Being less focused on a specific technology, they shift the perspective to its implications. Placing the fiction in the future or an alternative world helps to make this shift. In our study, we developed four design fictions: written stories describing a day in the life of four fictional characters living with slightly different smart home systems. All four systems included the following aspects taken from cultural probing:

- Energy awareness and use, e.g. energy feedback, comparison with other households, advice on energy saving, master on/off switch
- Home Automation, e.g. data access, sensor readings access, automated control of home parameters, remote control of home parameters
- Communication, e.g. messaging system within a building complex, (virtual) bulletin board, community events
- (Home-) Organization and Information, e.g. synchronizing calendar data with feedback and automation, notifications

**Fig. 2:**
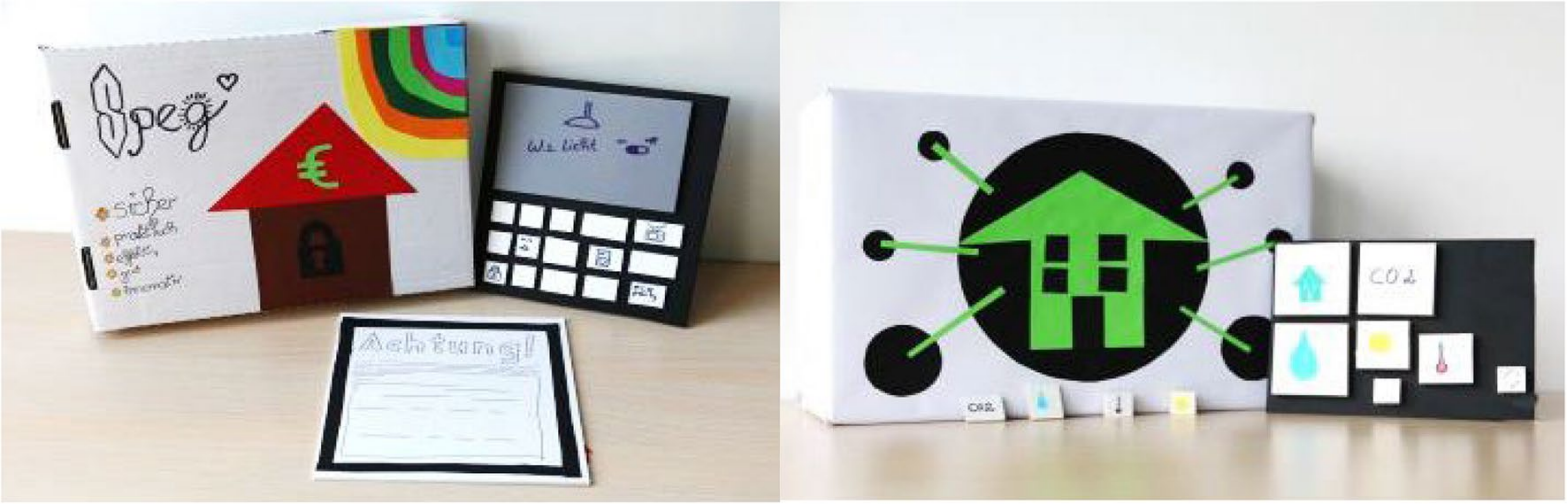
Example product boxes and system mock-ups from design fiction workshops

The design fictions were used in a series of four workshops with a total of 34 participants (Table 2). The workshops first elaborated on the design fictions through a SWOT-type analysis (Hohmann 2006), identifying strengths, weaknesses, opportunities and concerns (renamed from “threats”), which were followed by two rounds of physical prototyping. For the SWOT analysis, participants formed groups of two to four members. Each group selected one of the four fictions, performed the analysis, and discussed the identified aspects with the other groups. After collecting all aspects, each person distributed five points among the collected aspects to indicate their relevance. The groups then reformed and selected four aspects to elucidate which positive or negative consequences these aspects would have on people’s lives and smart home design, and how they could be avoided or ensured. The first physical prototyping drew on the innovation game “Product Boxes” (Hohmann 2006), which aims at generating key characteristics a future product should have. The groups were asked to imagine an ideal smart home system that would implement, extend or avoid aspects discussed earlier and design its packaging using materials like cardboard boxes as if it was readily available in a store. Through this, participants did not only collect features but also imagined how the qualities of the product would be advertised. In the second round of prototyping, the groups used sandwich panels and other crafting material to create physical mockups of their system. This aided in sharpening their vision of the smart home, it’s physical integration into the home and enabled a contrast between the actual system and promises made by its advertisement. The groups presented their product boxes and systems to the other groups with ample space for discussions (Fig. 2).

**Table 2:** Design fiction participants

Gender	
Male	16
Female	18

Age group	
19–35	6
36–50	9
51–64	6
65+	3

Interest and commitment (very – more – less – not interested/committed)	
Technology	22 – 5 – 5 – 2
Environment	24 – 8 – 2 – 0
Community	12 – 14 – 8 – 0
Politics	6 – 11 – 11 – 6

Education	
Compulsory education	3
Apprenticeship	6
Vocational school	6
Secondary school	9
College/University	10

Living situation	
Single	12
Domestic partnership, children	6
Domestic partnership, no children	10
Flat share	5
Other	1

### Design and evaluation of smart home components

**Fig. 3:**
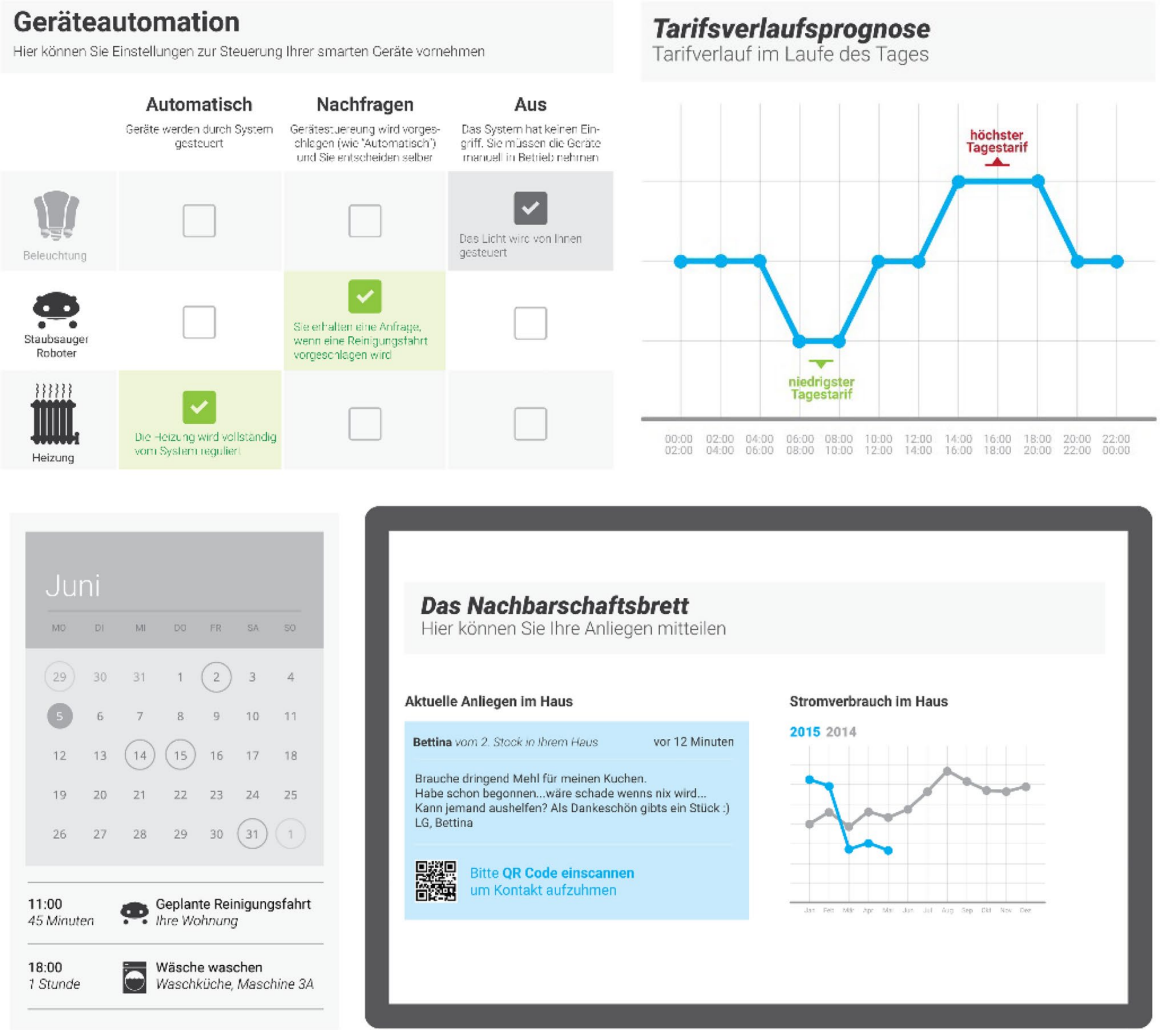
Four design mock-ups: an aspect of home control—settings for lights, vacuum-cleaner and heating (top left), information about the current tariff (top right), a household calendar (bottom left) and a public display (bottom left)

The third phase translated the high-level design fictions into tangible design solutions, which were evaluated and improved involving potential users. In a first step, we analyzed the results of the design fiction workshops and identified four key aspects: system characteristics, features, main concerns, and mitigating factors. A professional user interface designer created interface mock-ups for each of the emerging aspects to serve as a discussion basis in focus groups. The mock-ups were used as a conceptional anchor rather than a final product—to provide a low-fidelity and easy to grasp version of an aspect. Discussion was subsequently steered to elucidate the underlying idea rather than its implementation. The individual designs (Fig. 3) did not form a complete system, but were combined into three booklets according to their main theme:

- *Communication and information* included a digital neighborhood blackboard, a discussion forum, a personal messaging service, a petition support platform, a public display, a local newsletter, and a real-time public transport departure monitor.
- *Control and automation* comprised of (automatically) controlling various appliances, e.g. lights, machine learning from user behavior, remote access and notifications.
- *Energy feedback and organization* included energy consumption feedback and notifications, a flexible tariff, a digital shopping list and a household calendar.

We hosted four focus groups with a total of 26 participants (Table 3). The focus groups consisted of a walk-through of all three booklets. Participants first browsed a booklet, annotating it individually. We then opened the discussion, using a set of guiding questions per screen, and letting the discussion develop before moving on to the next booklet. The questions were based on the results of previous phases and covered aspects of type of use (e.g. “In which situations do you want manual control?”), information needs (e.g. “What type of energy data do want to see here?”), willingness to share or use (“Would you be willing to share this with others?”), expected benefits (e.g. “Do you think this would save you time or make your daily life more comfortable?”) and potential concerns (“What do you think happens if the system breaks down?”).

**Table 3:** Focus groups participants

Gender	
Male	15
Female	11

Age group	
19–35	10
36–50	12
51+	3
65+	1

Interest and commitment (very – more – less – not interested/committed)	
Technology	21 – 2 – 3 – 0
Environment	22 – 3 – 1 – 0
Community	12 – 12 – 2 – 0
Politics	6 – 7 – 7 – 6

Education	
Apprenticeship	6
Vocational school	3
Secondary school	10
College/university	7

Living situation	
Single	8
Domestic partnership, children	4
Domestic partnership, no children	10
Other	4

### Analysis and categorization

In the following, we give an overview of the data-streams resulting from the research phases, how they were categorized and synthesized.

**Fig. 4:**
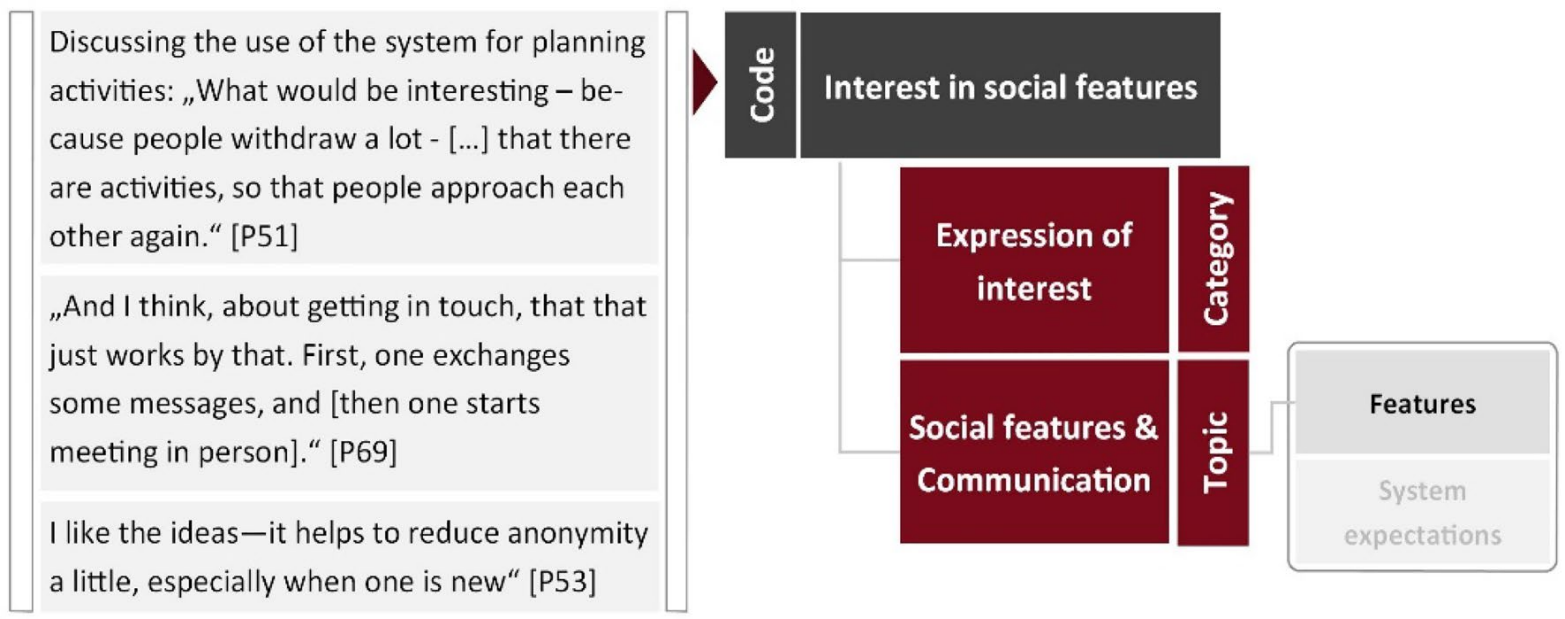
An example of how statements (translated) were coded, categorized and clustered

*Cultural probing* yielded photos, diary entries, and interview statements, which were clustered to identify their main topic. Being concerned with the status quo in homes, they enabled insights different from the workshops or focus groups. The *design fiction workshops* provided aspects identified and rated in the SWOT analysis, product boxes and system mockups, and discussion statements. SWOT topics were sorted, and scores consolidated across workshops. Product box and system mockup descriptions were separated into type of product (e.g. software, in-home monitor), control devices (e.g. dedicated remote), controlled appliances (e.g. heating, light), features (e.g. assistive functions), attributes (e.g. small, safe), anticipated consequences (e.g. growing lonely) and miscellaneous items (e.g. function-wise limitation of system internet access). Discussions in the *design fiction workshops and focus groups* were partitioned into individual statements. Each statement was coded according to the features, system abilities, mental models and expected consequences it contained. After compilation, each code (e.g. interest in remote control) was classified according to its main topic (e.g. home control) and category (e.g. expression of interest), see Fig. 4.

## Findings: desired features and system expectations

**Fig. 5:**
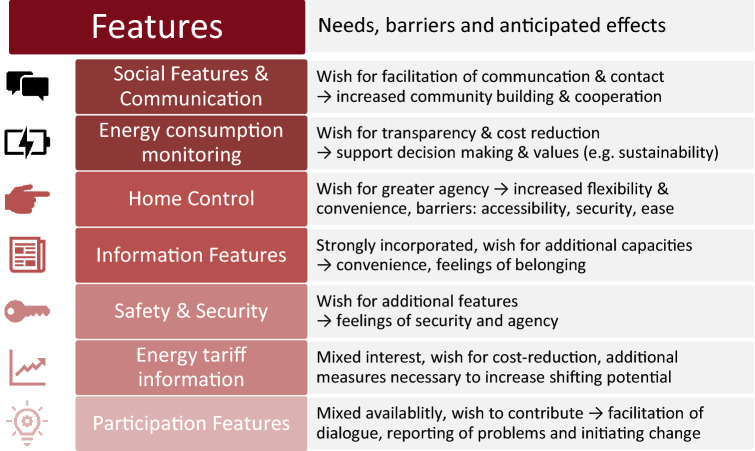
Features with brief overview of main needs, anticipated effects and barriers, already showcasing some of the emerging design dimensions, like accessibility and ease of use

**Fig. 6:**
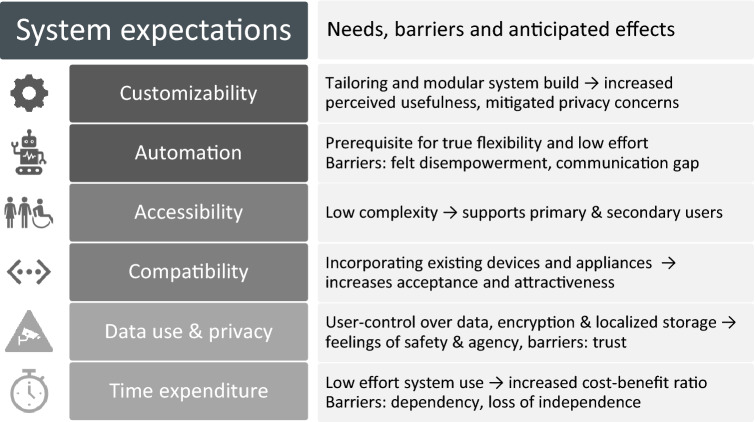
System expectation with brief overview of main points and anticipated effects

Two categories of main topics emerged from the analysis of the code: *Features* (4.1, Fig. 5) refers to seven specific areas of operation or modules of the envisioned smart home system that were introduced through in the probing package, the design fictions or the design screens (e.g. energy use) or came up during interviews, workshops, and focus groups (e.g. safety and security). *System expectations* (4.2, Fig. 6) were elucidated mainly through discussing and developing features and refer to more general system properties including expected consequences of system use and system abilities.

### Features

*Social features and communication* was the most diverse feature including 27 different subtopics discussed on 5 categorical levels. Cultural probing highlighted both current practices of communication and well as a wish for extending them. It was among the top five rated SWOT topics and one of the most discussed topics in the focus groups (over 40 discussion instances). Participants were deeply interested in combatting anonymity (yet preserving privacy, 30 discussion instances), facilitating the initiation of contact and providing an easy means of communication within apartment buildings. It was most important to participants that the features would not be designed to replace face-to-face contact (16 discussion instances) but enabling it. They expected that such features would increase community building and neighborly cooperation, assist in organizing activities or use of communal rooms, though some participants also voiced concern a potential misuse of such a “network” (e.g. surveillance, bullying, and presence-tracking).

*Energy consumption monitoring* was also a diverse subject, discussed on four categorical levels and 21 subtopics, including cost transparency and cost reduction, which were two of the top five rated topics in the SWOT analysis (7 points each). Additionally, energy feedback was most strongly spoken for (16 discussion instances voiced interest). Through all three phases, this interest was largely based on financial considerations (possible savings or rewards), personal attitudes such as energy-saving as value, transparency as a need, sustainability as a personal goal, or economy/austerity as a moral good. Participants considered access to individual information as necessary to support or attain those goals. To them, the purpose of energy monitoring technology was increasing the intelligibility and transparency of energy use and costs, and empowering users to make informed decisions. Participants were interested in energy as well as water consumption. They noted the importance of visualizing costs as well as consumption units, a certain amount of accessible detail (breakdown to appliance-level or activities) and forecasting capabilities. Less popular options were normative comparisons, energy consumption predictions including new devices and the ability to compare energy suppliers. Participants’ greatest concern was consumption monitoring being time-intensive and therefore only feasible for a limited time. They, therefore, preferred ambient information for continuous long-term monitoring.

*Home control* included 15 different subtopics on two categorical levels (feature description and expression of interest). Remote control was the most discussed single feature by far (28 discussion instances) and among the top five rated topics in the SWOT analysis (7 points). Participants expressed a need for increased flexibility and convenience, which they would realize by adapting home control to individual routines and circumstances. Participants made a distinction of technology “embedded” in the home (e.g. lighting, heating) and appliances (e.g. washing machine, personal devices), connotated by a different emotional attachment, which nonetheless should both ideally be remotely controllable via app, SMS or web interface. Participants’ concerns included reduced accessibility for people without smartphones, data-tariffs, technological know-how, or capacity. Other barriers were perceived security issues, the unclear general setup (e.g. contract and legal conditions), and its use in multiple user homes. They mentioned the necessity of automation, appliance programming, and fine-grained control-models (e.g. rooms, appliances) to use smart home control in a real-life setting.

*Information features* were already strongly incorporated in households taking part in the cultural probing. Participants employed different surfaces to share mobility information, information about the surrounding area, timetables and individual information. In a virtual environment, they would additionally store household information (e.g. device manuals, shopping support e.g. light bulb types). Participants noted that sharing regional information encourages community building and feelings of belonging.

*Safety and security* contains features for the living space (seven subtopics) and assisted living (four subtopics). The former included alerts of trouble within the home (e.g. fire, water damage) and other features that would make participants feel safer, like burglar deterrents (light automation and remote access), physical access control, and door/window sensors. The latter discussed features like assistive home control and household monitoring by family members. Their feelings of security were heightened by features concerned with support in emergencies as well as by the general overview of one’s household, appliance use and energy consumption.

*Tariff information and transparency* were expressed as important features, especially with a time-variable tariff. Participants equated a variable tariff with a time-of-use tariff (i.e. fixed time slots of different pricing), which was seen as both potentially positive and negative—as an opportunity for transferring “favorable price-realities” to consumers as well as for increasing overall costs and controlling individual energy consumption by energy providers. A need for comfort, which many participants were not willing to compromise within their own home, was a major barrier. Participants noted that the potential to shift activities would be small, and the time and effort in managing consumption would largely exceed the savings, which were perceived negligible. They did note that shifting potential could be increased if an energy monitoring system allowed remote access to appliances and automating domestic actions. Another mitigating possibility was temporary energy storage capacities (e.g. batteries) in the home.

*Participation features* Within the cultural probes and succeeding interviews, participants described that they frequently notice problems in their immediate environment and have ideas for improvement. While some participants stated they have satisfactory possibilities to become active, others felt dialog was currently not properly facilitated to hear their complaints or realize changes. Smart home features that enable participating, contributing to initiatives, as well as simple reporting of problems encountered in the building or surrounding area, were met with interest by these participants.

### System expectations

*Customizability* was a main expectation, answering to the perceived poor fit of systems in the cultural probing phase: tailoring features (4 SWOT points, 9 discussion instances) and modularly creating systems mitigated many perceived privacy issues and increased the perceived usefulness of a smart home. Participants’ choices were strongly related to their individual level of comfort with technology and need for control within the home. Within all phases, participants expressed divergent goals—e.g. a wish to gain increased awareness and transparency as well as support for different values (e.g. sustainability, economy, privacy). They, therefore, perceived a system as valuable only if it specifically catered to their needs and accommodated these personal goals.

*Automation and intelligent learning algorithms* were a prerequisite to realize true flexibility in participants’ minds—especially if a system includes time-variable tariffs. On the other hand, others noted that automation and learning behavior could disempower users, creating a communication gap between them and their homes and thereby reducing home comfort as well as increasing surveillance and individual “transparency”.

*Accessibility* Imagining their ideal systems, participants expressed a need for low complexity, which would make it accessible for varying user groups. Systems should cater to people with less technical knowledge and experience as well as people with disabilities by including specific, user group-oriented designs and support. Participants also discussed secondary users, such as persons visiting equipped households (e.g. grandparents babysitting their grandchildren) that need to be able to interact with the system.

*Compatibility* with existing devices and home appliances was another factor for the acceptance and attractiveness of a smart home—the necessity to refit an entire household in introducing such a system was a great barrier and possibilities for extending to other areas like car-sharing and other applications already in use highly desirable.

*Data use, privacy and unauthorized access* Participants had little trust in data protection measures provided by energy providers or connected facilitators. They were therefore skeptical to connect their (imagined) home to the Internet or other networks. While some saw this as unavoidable and resigned themselves to the cost, “paying” for services with their data and privacy, others called for settings allowing detailed control of data-transfer, appropriate data encryption and the localized storage of (critical) data. Concerns related to privacy loss included surveillance by energy providers and other third parties, but also monitoring by other household members. That their daily actions within their own homes might become traceable was unimaginable to some participants and irreconcilable with their needs. This traceability and possible data misuse received eight SWOT points each, while the “transparent customer” received 10 SWOT points. The concern of unauthorized access acted as a further barrier to remote control features.

*Reliability* was met with skepticism. Participants did not perceive current technologies as stable and usable enough to entrust them with their homes and insisted on local and manual control as a fail-safe if the system is unresponsive or connection is lost. They questioned the (hidden and uncommunicated) costs of support and noted that liabilities might not always be clear (e.g. what would happen in the case of data theft or unauthorized access).

*Time expenditure and long-term use* were both framed negatively: participants assumed that system use would generally lead to a higher effort to manage their lives, especially with time-variable tariffs, rating excessive planning of daily lives as one of their top five concerns (2 SWOT-points and 5 discussion instances). This high system cost (personal energy, money, time) was contrasted with comparably small benefits over time, also noting several negative consequences of long-term use. Of these, dependency on the system and technology, “dumbing down through technology use” and loss of independence were the most important subjects (12, 8 and 3 SWOT points, 2, 8 and 10 discussion instances respectively). Yet, they also noted that high system costs were based on bad design, namely the overall poor usability of home technology and technology-centrism of systems, and that clear, sustained benefits could make time-intense processes more attractive (e.g. financial incentives).

## Design implications

Based on these results we developed recommendations to inform the design of future smart home solutions. We will first outline a *practice-based and value-centered design process*, before discussing specific *design dimensions* and *design goals*.

### Designing with (smart) home practices and values

**Fig. 7:**
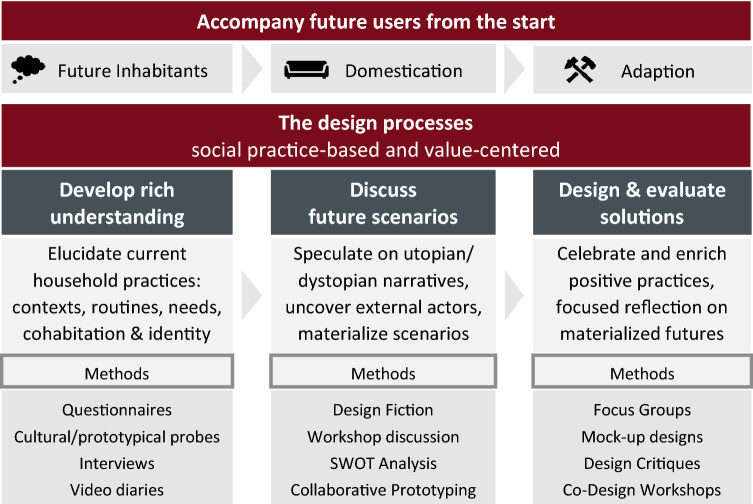
Moving beyond technology acceptance or user requirement models is a deeply participatory process: accompanying future users from the start can help cover stages of domestication and adaption of technology. Implementing a design process which is based in social practice and centered around values spans three stages with different foci and might employ a number of different methods (lists in this illustration are non-exhaustive)

In what follows, we outline a social practice-based and value-centered design process for smart homes (Fig. 7). Such a process has to be deeply participatory in order to reflect a plurality of human and nonhuman (e.g. environmental) values and avoid an agenda driven by techno-fix utopias or corporate growth interests (Strengers 2016). Ideally, the process begins with future smart home inhabitants and continues into phases of domestication and adaptation (Mennicken and Huang 2012a; Hargreaves et al. 2018). In the reality of time-bound research contexts, this might be challenging, as project schedules for design and building construction frequently misalign, particularly if there are construction delays, as we have seen in this study. Still, designers need to work flexibly with available participants to avoid designs being decided by other stakeholders. Of course, in complex, multi-actor projects with considerable financial investments, which smart home projects often are, the inclusion of a participatory design process for smart home technologies does not necessarily mean that results are being implemented, even if done on time (e.g. Bødker and Zander 2015). Powerful (corporate) actors can disregard them as much as they promote other technologies into the home (e.g. smart metering) because they serve their commercial interests. As such, working with future renters and owners is, if possible, even more important, to increase legitimacy of the outcomes when negotiating with technology providers.

The process as outlined in the methodology proved successful in moving beyond simplistic technology acceptance or user requirement models and develop a rich understanding for domestic practices and values, generate differentiated narratives to stimulate critical reflections, and specific design goals and dimensions (e.g. Mäyrä et al. 2006; Haines et al. 2007). Cultural probing and the follow-up interviews allowed us to start a conversation with our participants that was not centered on functional requirements or a solution to ‘fix’ or ‘improve’ unsustainable or inefficient behavior. Instead, we began with understanding current material practices at home, such as household routines, household needs and cohabitation practices. Starting open, the design process avoided to pre-conceive corrective technologies, but rather to celebrate positive practices (Grimes and Harper 2008; Ferdous et al. 2017). For example, many participants had a high awareness of their energy use, and established practices for optimization, which should rather be transformed and enriched by technology than disrupted. While the probes allowed participants to respond and reflect in their own time without researchers intruding private space, they formed useful prompts to discuss domestic practices in more depth. For instance, several households had established spaces that served as hubs for information sharing and communication. Finally, the probe and interview data informed the writing of the design fictions both in the choice of contexts as well as for issues individuals might be confronted with. The probes specifically highlighted the variety of routines individuals structure their lives with, and how these impact their homes. They added context factors that shape energy and device use, showcased how participants implemented values and identity into their home and added a spacial dimension to relations within and around the home. This acted as a back-drop to speculate on technology effects in the design fictions. Besides cultural probing, we can imagine that this part of the design process could be enriched by more dynamic ethnographic methods, such as video diaries (e.g. Keller et al. 2008), or through low-fidelity smart home prototyping: if participants could take home provocative mock-ups of smart home technologies and explore through everyday interaction, discussions and speculations among household members, it would uncover deeper layer of how such technologies fit into or disrupt their lives, practices and value systems.

The design fictions moved the discussion from current practices and values to the future, but again, instead of narrow functional requirements, they offered an open space for smart home speculations and deliberately included ‘utopian’ and ‘dystopian’ narratives. As we showed in our analysis, this facilitated uncovering external actors that shape home environments, for example, the anticipated actions and motives of energy and technology providers. Including a main protagonist in each story additionally allowed individuals to identify with them and/or to contrast their own individual needs and circumstances with the narrated story. The SWOT analysis was useful for collecting an overall picture of both positive and negative aspects of the smart home speculations, to elaborate on these aspects and to discuss and agree upon priorities. Working in a different mode, the product boxes and system mock-ups supported participants in *materializing* smart home futures. While playful, they were tangible objects of desires, needs, concerns, and critical reflections. For instance, they foregrounded the contrast of what a smart home promises and what it could actually mean, critically appraising the technology in a very lighthearted manner, something that could not be captured by a SWOT analysis. In this study, we created four design fiction narratives with a graphically designed backdrop as workshop prompts, which is a common way of using design fictions (Blythe and Wright 2006; Linehan et al. 2014; Huusko et al. 2018). It would, however, be interesting to explore more participatory forms of design fiction creation, as suggested by Prost et al. (2015) and as initiated with the product boxes and system mockups. The granularity of these two particular formats is limited, which was necessary in the context of a 3-h workshop. Working with participants in a more extensive setting could include participants writing their own preferred or feared design fictions or using role-play or video-making to enact their visions. This has for example been explored by Baumann et al. (2018) and Newell et al. (2006) and as they show, can bring rich and nuanced futures to the fore. Considering the increased personal investment (time, energy), working with people who will ultimately benefit from their participation because they will live in a smart home that they co-designed, becomes even more important.

The rich narratives generated in the design fiction workshops reflected the varied practices and values of our participants and thus provided a base to develop specific design mock-ups whose features respected and supported these practices and values. Moving from general narratives to more focused discussions, the format of a focus group provided a productive platform for critical evaluation of and reflection on the design proposals to develop specific requirements for implementation. Analyzing the focus group data allowed us to translate the design booklets, which were centered around the core themes of the design fictions, into specific *design dimensions* and *design goals*. We will discuss the dimensions and goals in detail in the next sections; here we want to draw attention to how the focus group enabled this translation work. For example, the booklet design of energy information was discussed in the focus group as enabling action-taking in line with individual goals (sustainability or financial savings respectively) and whether these could be reached by intervention, thus representing the design dimension values and the design goal of benefits. Similarly, the booklet design on automated household control was reflected through the value of taking manual action. Actions, in this sense, represented more than, for example, the turning of a switch, but confirmed and reinforced the shared set of values that also give a household social coherence. This dialog formed essential parts of the design dimensions of relations and values. Focus groups are an established method, suited to both efficiently cover set topics (such as the different interface designs), but are also flexible in that they allow the participants to drive the conversation (Morgan 1997). Besides focus groups, we can also think of other formats, such as design critiques, which would enable a more extensive conversation about design intentions, methods, functions, materials, business models, meanings, human needs, implied users, and experiences (Blevis et al. 2007; Bardzell 2018) Alternatively, one might want to enable more explicitly participants to co-design interaction solutions. Instead of the professional designer designing prompts, a half or full-day co-design workshop (e.g. Huusko et al. 2018) or hackathon (e.g. Thomer et al. 2016; Taylor and Clarke 2018) could be facilitated by designers and include technology experts as consultants. Using, for example, design fictions as starting points, participants could use a range of craft materials, software and hardware to build interaction modes, interface mock-ups, and prototypes. This would position users as experts of experience, not just in the role of evaluators of design, but also as designers and creators with ownership over and identification with the home they design.

**Fig. 8:**
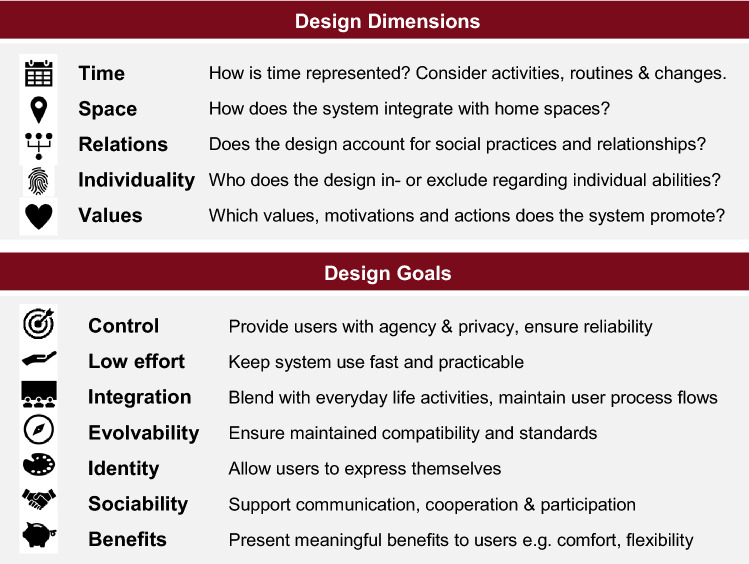
Recommendations to inform the design of future smart home solutions: *design dimensions* and *design goals* show what design should acknowledge and aim for

Co-design workshops and hackathons then form logical connection points to carrying on active involvement of future smart home dwellers into the actual implementation and construction of smart homes. Through participatory and agile software development processes (e.g. Chamberlain et al. 2006; Hansson et al. 2006), participants, with or without prior technical knowledge, can either be kept ‘in the loop’ to provide continuous feedback and evaluation, or can learn to program, build, customize, or hack their own smart home technologies (e.g. Kautz 2010). In the context of this study, the detailed design and implementation phase is outside of the scope of this paper. However, we now want to position the design dimensions and goals derived from the focus groups that will frame further detailed design and implementation (Fig. 8).

### Design dimensions

Design dimensions address background factors designers should consider to complement the more system-centric “user context” (Solaimani et al. 2013a): dimensions apparent in this study, in line with (Davidoff et al. 2006; Taylor et al. 2007), are time, space, relations, individual factors, and values.

*Time* means designing in the context of activities, routines and household evolution as well as dealing with exceptions and change. To design along the dimension of time means asking whether a design allows for temporal expression of the home context, and how time is (re-)produced in a system (e.g. schedules).

*Space* addresses designing for spaces with specific functions and meanings. To design along the dimension of space, designers should ask how a system interacts and integrates with spatial dimensions of the home, where it is located and whether it creates new locations or spatial links.

To design along the dimension of *relations,* design must acknowledge multiple users, shared ownerships within the home, and negotiation of competing needs and varying levels of comfort. A system designed for relations must additionally account for social practices and relationships constraining as well as enabling actions (e.g. primary responsibility for domestic labor) (Murtagh et al. 2014).

*Individual factors* focus on additional individual characteristics and abilities (e.g. computer literacy). Systems can be viewed by the in- or exclusion of factors, and thereby, of people, in their design. Designing along this dimension therefore also addresses users’ concerns about smart technologies excluding specific groups (this study, Balta-Ozkan et al. 2013b).

*Values* address feelings, motivations, views and goals that frame actions and activities in the home context. Designing along this dimension, therefore, includes consideration which values, motivations and actions a system chooses to promote and how they relate to pre-existing value structures. While values are individual, they are also shaped by the larger cultural context: similar to US and UK studies (Davidoff et al. 2006; Taylor et al. 2007), our participants reported emotional connections to their home, yet they saw it less as a product, whose value is increased via smart features (Davidoff et al. 2006). Similar to participants from the UK (Balta-Ozkan et al. 2013b, 2014), our participants held specific views on trust, privacy and security, which evoked strong, emotional responses. Like participants in the UK and Finland (Karjalainen 2011; Balta-Ozkan et al. 2014), many of our participants were environmentally concerned and motivated by this value. While “busyness” was noted as a “moral good” in a study from the US (Davidoff et al. 2006, p. 26), we identified “being economic” and “being self-sufficient, not passively relying on a system” as core values that need to be addressed to prevent negative emotions of guilt and worry (e.g. about “becoming lazy” and unable to function without a system). The notion of “becoming lazy” was also noted in a UK study (Hargreaves et al. 2018). Jensen et al. (2018b) showed how values can be effectively co-addressed. In their probing study of two Australian households, an aesthetic concept of comfort successfully but indirectly promoted sustainable energy use.

While these design dimensions can be viewed separately, they are in fact intersectional: for example, negotiation was important for all non-single households, combining the use of specific spaces for information storage *(space)* and the practice of sharing (*relations)*. Similarly, the importance of routines (*time*) (this study; Balta-Ozkan et al. 2013b) is reflected in *values* when users fear apathy or “losing” such household routines. Embedded in routines (*time*) is the interdependence of individuals (*relations*), which might vary with societal context. Our participants, for example, reported less general interdependence of parents and children due to different mobility and enrichment activities than US-based homes did (Davidoff et al. 2006). Thus, design along one dimension should acknowledge its effects on another and take its prerequisites into account.

### Design goals

In contrast to design dimensions, design goals provide aspects design should aim for. Based on the collected material we derived seven design goals:

*Designing for control* refers to control of lives rather than control of systems (Davidoff et al. 2006) and to homes being places of control (Innocenti 2017), meaning agency, efficacy and decision-making authority rather than managing settings and devices. Designing for this kind of control should encompass felt agency, system reliability as well as control of data use and privacy. Privacy and data security were often framed as issues of choice and control. Participants saw smart home systems as vehicles for increased agency through knowledge and transparent information, yet also anticipated loss-of-control feelings (e.g. “over-automation”) and actual loss of control (e.g. system malfunction). This mirrors privacy concerns across China and Europe (Balta-Ozkan et al. 2013b; Zhai et al. 2014; Deloitte Consulting and TU München 2015): Storage and eventual use of personal information (selling, use in advertisement) were as much issues as monitoring with third parties potentially knowing daily routines and home occupancy. Designing for control could also include low entry options for collaborative development of smart home technologies, which could include passive users in homes with more technologically interested individuals without burdening them with direct system development since especially for this group, gains in comfort was counteracted by felt loss of control (Mennicken and Huang 2012a).

*Designing for low effort* focusses on low time expenditure and practicability. This can be achieved through various means, including system control and user-system communication. Of these, automation can be one, though barriers in the form of technological immaturity, low intelligibility and impractical user control persist. Low effort has been especially discussed in connection to time-variable tariffs: similar to previous studies our participants were hesitant about their use and practicability (Karjalainen 2011; Balta-Ozkan et al. 2014).

*Design for integration* with everyday life practices takes a slightly different angle on designing for control and designing for low effort: people spend a substantial amount of their time at home, engaging in different activities and practices. Everyday activities are the focal point of what it means to be ‘at home’. Integration with everyday life practices thus focuses on integrating smart functionalities with everyday practices and routines, without interrupting process flows or forcing users to shift their focus from the tasks at hand towards details of system control. Smart functionalities should offer additional possibilities that support users in achieving their goals, but not require them to modify their behavior. They should furthermore aim to minimize gaps and discrepancies in media usage (e.g. the need to switch between devices), physical location (e.g. having to go to another room to access the control point) or atmospheric dispositions (e.g. need to interact with a very demure control interface in an entertainment context).

*Designing for evolvability* includes learning as well as maintained compatibility*.* While designing for initial compatibility is an important system component and architectural consideration, maintained compatibility is integral for system evolution. From a system’s point of view, evolvability can be noted as one component of a system’s agility (Solaimani et al. 2013a). Only evolvable systems can reflect changing household needs or changed circumstances such as children entering school. Maintained compatibility also ties in with the development of the field of smart homes: developing standards for system languages and operating systems would not only ease adding capabilities and devices when needed but also, as Mencken and Huang (2012a) suggest, support capable users in developing their system and minimize negative impacts on other user groups.

*Designing for identity* is especially relevant for the home context: home fulfills important functions in enabling and expressing identity besides the more mundane function of providing shelter and living space. People use their homes to express and ‘materialize’ their identity (this study; Davidoff et al. 2006; Innocenti 2017). Smart home concepts should support these practices and different ways of self-definition (including non-technical ones), especially in the view of enabling appropriation and sustainable smart home development.

*Designing for sociability* including communication, cooperation, and participation, should be one of the main design goals to support sociability and participation as values: active decision making, partaking of communal development and communication proved important personal motivators for our participants in the specific context of an urban environment. It remains to be investigated if this topic is of importance in different settings and how well technology might integrate with “value lines” in the social context. We also note that interest in communication, activity and participation have strong social desirability, possibly biasing contributions to this topic.

*Design for benefits.* In the smart home context, benefits include added comfort, increased convenience, security and flexibility. Benefits need to compensate for costs—in this study as well as in Balta-Ozkan et al. (2013b) these related to installation, repairs and maintenance, vulnerability to rising prices and non-monetary costs (data and privacy as currencies). Yet, design for benefits should generally aim higher than mere acceptance of a system or counterbalancing perceived and actual costs. Even when benefits are perceived, they are on the scale of small conveniences and the facilitation of individual tasks but neither substantially support users, nor enrich their lives in a meaningful way (Mennicken and Huang 2012a). Design for benefits should address users’ perceived lack of benefit (this study; Balta-Ozkan et al. 2013b; Deloitte Consulting and TU München 2015) and specific beneficial features punctually, but moreover needs to include real, tangible, and significant benefits to user’s lives *by design*. Features beyond the traditional realm of smart homes, in particular social, informative and participative features potentially increased the value of systems for participants in this study. Studies have shown that approaches highlighting inhabitants’ desires, motivation and aesthetic needs can be successfully combined with desired functionality and desired outcomes—and that viewing smart homes through this lens enables design capable of both involving “dumb” and non-electrical aspects of the home and contribute to its meaning and potential benefit (Jensen et al. 2018a, b).

## Conclusions

This study describes one of currently few attempts to integrate contextual user requirements analysis with application-oriented design thinking. Such an integrated approach is necessary to leverage the advancement of the ‘Smart Home’, which has so far been driven more strongly by technology and business push than by genuine user demand. Viewing the home as an individual everyday environment has enabled us to identify design directions that achieve a closer fit between design and context. Based on extensive empirical data we identified important features and system expectations relevant to users. From this, we derived design dimensions and goals that can help developers to understand the context of use and provide decision support for the prioritization of design directions.

The study further contributes with a methodological concept that encompasses three successive steps: cultural probing, participatory design fiction and focus groups. To our experience, this specific qualitative research process fulfilled the goal to progress from an understanding of the home context “as-is” and critical speculation on smart home futures towards a blending of requirements with technological solutions. Future research should investigate optimizing the effort and execution timeframe of such combined qualitative approaches, while still maintaining the richness of contextual design insights. This would make this approach even more applicable, especially for smaller projects in commercial development settings.

Our findings provide a comprehensible framework for the design landscape for smart home systems and serve as a basis for integrating previous findings into an overall framework. Subsequent research should explicitly build on the design dimensions and specific design goals we derived. The focus for such further investigations should be to assess their actual value in a design process, in order to develop them further with regard to completeness and relevance for implementation work.

## Data Availability

Aggregated and anonymized data only.

## Footnotes

1 *Hygge* denotes “a quality of cosiness and comfortable conviviality that engenders a feeling of contentment or well-being (regarded as a defining characteristic of Danish culture).” (Oxford Languages).
